# The effect of sacrospinous ligament fixation during vaginal hysterectomy on postoperative de novo stress incontinence occurrence: a prospective study with 2-year follow-up

**DOI:** 10.3906/sag-2005-117

**Published:** 2020-06-23

**Authors:** Eralp BAŞER, Kerem Doğa SEÇKİN, Pınar KADİROĞULLARI, Hüseyin KIYAK

**Affiliations:** 1 Department of Obstetrics and Gynecology, Liv Hospital Ulus, İstanbul Turkey; 2 Department of Obstetrics and Gynecology, Faculty of Medicine, İstanbul Health Sciences University,Kanuni Sultan Süleyman Training and Research Hospital, İstanbul Turkey; 3 Department of Obstetrics and Gynecology, Faculty of Medicine, Acıbadem University, İstanbul Turkey

**Keywords:** Pelvic organ prolapse, stress urinary incontinence, vaginal cuff prolapse, sacrospinous ligament fixation

## Abstract

**Background/aim:**

To investigate the risk of de novo stress urinary incontinence (SUI) occurrence in women who were treated for pelvic organ prolapse (POP) with sacrospinous ligament fixation (SSLF) in addition to vaginal hysterectomy (VAH) and antero-posterior colporrhaphy (CAP) over a 24-month follow-up period.

**Materials and methods:**

A prospective randomized study was designed. Women without occult or obvious SUI were randomized into either one of the study groups: Group 1: VAH + CAP, and Group 2: VAH + CAP + SSLF. Postoperatively, the patients were reevaluated for de novo SUI occurrence.

**Results:**

A total of 150 women were analyzed [G1 = VAH + CAP (n: 77) and G2 = VAH + CAP + SSLF (n: 73)]. Mean age, parity, body mass index, menopausal status, and preoperative POP degree, grade 1 and grade 2-3 cystocele and rectocele frequencies were similar between the 2 groups. During follow-up period, de novo SUI developed in 7 patients (9.1%) of Group 1, and in 6 patients (8.2%) of Group 2 (P > 0.05). In Groups 1 and 2, POP recurrence occurred in 5 (6.4%) vs. 1 (1.3%) cases,respectively (P < 0.05).

**Conclusion:**

In patients undergoing surgery for POP, the addition of SSLF did not result in an increased rate of de novo SUI. Careful patient selection, and informing the patients about the risks and benefits of the planned surgical procedure are essential steps in each case of POP.

## 1. Introduction

Pelvic organ prolapsed (POP) is a very common condition, approaching nearly 50% in middle aged women [1]. POP is corrected surgically in many of the cases. POP degree, menopausal status, patient and surgeon preference are the most important contributors on final treatment choice. Vaginal hysterectomy (VAH) is commonly selected, and colporrhaphy is frequently performed as a part of this procedure in patients who have completed childbearing [2]. Stress urinary incontinence (SUI) is defined as involuntary leakage of urine following a sudden increase in intraabdominal pressure, which can be the most evident during activities such as straining, coughing or laughing. Occasionally, de novo SUI might occur after POP surgery, and further might even require additional surgeries [3]. Previously, rates of de novo SUI after POP surgery have been reported between 9.9% and 51% [4–11].

In patients with POP in whom a VAH is planned, the addition of sacrospinous ligament fixation (SSLF) procedure might decrease the rate of postoperative vaginal cuff prolapse [12,13]. However, the addition of this procedure might also increase the operative time and the risk of perioperative complications [14–18]. The purpose of the present study was to determine the risk of de novo SUI occurrence in women who were treated with SSLF in addition to VAH and colporrhaphy anterior-posterior (VAH + CAP) over a follow-up period of 24 months. 

## 2. Materials and methods

### 2.1. Study design and setting

A prospective randomized study was designed following approval from the institutional review board (Approval number: KAEK 2016/2, Clinical trials: NCT03221725). Informed consent was taken from all study participants. The study setting was a gynecology and obstetrics department of a tertiary referral hospital in İstanbul, Turkey. This randomized clinical trial was conducted in compliance with the consolidated standards of reporting trials (CONSORT) guidelines. 

### 2.2. Participants

Between February 1st 2017 and February 1st 2018, eligible women aged between 45–65 years old that were planned to have a VAH + CAP due to stages 2 or 3 uterine prolapse and anterior/posterior compartment defects were randomly assigned into 1 of the 2 study groups. Study participants without symptomatic urinary incontinence (UI) were included in the study. The participants were allocated into either 1 of the 2 intervention groups. The participants in Group 1 underwent VAH + CAP, and the participants in Group 2 underwent VAH + CAP + SSLF.

Symptomatic UI was defined as UI that occurred at least once weekly and/or UI that was bothersome for the patient, and that scored “moderate or higher” according to Urogenital Distress Inventory-6 (UDI-6) [19]. All participants were also evaluated for objective signs of stress urinary incontinence (SUI) (cough stress test and urodynamic test) preoperatively, and only women without occult SUI were included. For all participants, cough stress test and urodynamic testing was performed with prolapse reduction. Additional preoperative exclusion criteria were the presence of medical comorbidities such as diabetes mellitus, morbid obesity (body mass index > 40), and history of prior vaginal or urogynecologic surgeries. A computer based randomization program was used for allocating the patients into either one of the study groups (Group 1 or Group 2) (www.randomizer.org). Postoperative exclusion criteria were major intraoperative complications (bleeding that required blood transfusion, bladder or bowel injury, prolonged urinary catheterization >1 week), and inadequacy of data due to loss during postoperative follow-up. Postoperatively, reevaluation visits on 6, 12 and 24 months were planned. During postoperative evaluations, urodynamic testing was performed only in patients who had positive cough stress tests. Participants who were lost to follow-up during these periods were excluded from the final analysis. The major aim of the study was to determine the occurrence of postoperative de novo stress incontinence (SUI). De novo SUI was defined as, postoperatively determined bothersome SUI that occurred at least 2 months after the initial surgery, lasting at least 3 months in spite of lifestyle modifications or urinary infection treatment, or requirement for an additional antistress incontinence surgery within the 24-month follow-up period. Bothersome SUI was identified with Urogenital Distress Inventory (UDI-6) [19]. The primary outcome measure of the present study was presence of all of the following: 1. Symptoms of urine leakage during coughing, straining, and/or Valsalva maneuver 2. A positive stress test during vaginal examination 3. A response of ≥2 on item 3 on UDI-6. Anatomical outcome measures were determined utilizing the pelvic organ prolapse quantification (POP-Q) system [20]. The total vaginal length (TVL) measurements were performed via physical examination. The difference between preoperative and postoperative measurements of TVL (ΔTVL), and urethrovesical prolapse (ΔAa) were recorded.

### 2.3. Surgical intervention and postoperative follow-up

In Group 1 (n: 85), VAH + CAP was performed, whereas in Group 2 (n: 85), VAH + CAP + SSLF was performed. As all of the patients had some loss of uterine apical support, a modified extraperitoneal uterosacral ligament suspension was routinely performed as a part of surgical technique in only VAH + CAP group (Group 1), in order to prevent future vaginal cuff prolapse. Postoperatively, patients were re-valuated on 1, 6, 12, and 24 months following surgery for de novo stress incontinence. Postoperative examinations were performed at the study setting, firstly evaluating the urinary complaints, and if any of complaints are present, performing UDI-6 questionnaire to determine severity of the symptoms. All patients had a vaginal examination, and cough stress test at all postoperative visits. 

### 2.4. Study data and statistical analysis

Patient data including age, parity, body mass index (BMI), menopausal status, past medical and surgical history, pelvic exam findings including POP-Q scores, preoperative and postoperative TVL and urethrovaginal prolapse change (ΔTVL and Δaa), uterine weight, operative time, preoperative-postoperative hemoglobin and hematocritchange (ΔHgb and ΔHtc), intraoperative and postoperative complications, and finally postoperative 6th, 12th, and 24th-month evaluations were recorded for final analysis. 

Statistical analyses were performed using SPSS 25.0 for Windows (IBM Corp., Armonk, NY, USA) . P values less than 0.05 were considered statistically significant. Categorical variables were presented as frequencies and percentages, and continuous variables were presented as mean ± standard deviation. The Kolmogorov-Smirnov test was used to test the normal distribution of all data. The Student’s t-test was employed for group comparisons of continuous variables with normal distribution. For comparison of nonnormally distributed continuous data, Mann-Whitney U-Test was performed. The chi-square test was used for comparison of normally distributed categorical variables. Sample size was calculated with an effect size of 0.85, alpha error 0.05 and power 0.80, a total of 62 subjects were needed (at least 31 subjects were required in each group). Setting the alpha error to 0.01 resulted in a sample size of 164 subjects (82 subjects in each group). Allocating 85 patients in each study arm was planned for the study. 

## 3. Results

During the study period, a total of 184 women were assessed for study eligibility. One hundred and seventy women were eligible, and were randomly allocated equally into one of the 2 study groups (Groups 1 and 2, n: 85 each) (Figure). Two women from Group 1 and 4 women from Group 2 had perioperative complications, and were excluded from the study. Furthermore, 2 women from Group 1 and 3 women from Group 2 were excluded due to prolonged urinary catheterization. During the postoperative follow up period, 4 women from Group 1 and 5 women from Group 2 failed to return for scheduled visits at the appointed times, and were designated as lost to follow up. Ultimately, 77 patients from Group 1, and 73 patients from Group 2 were available for final analysis (Figure).

**Figure:**
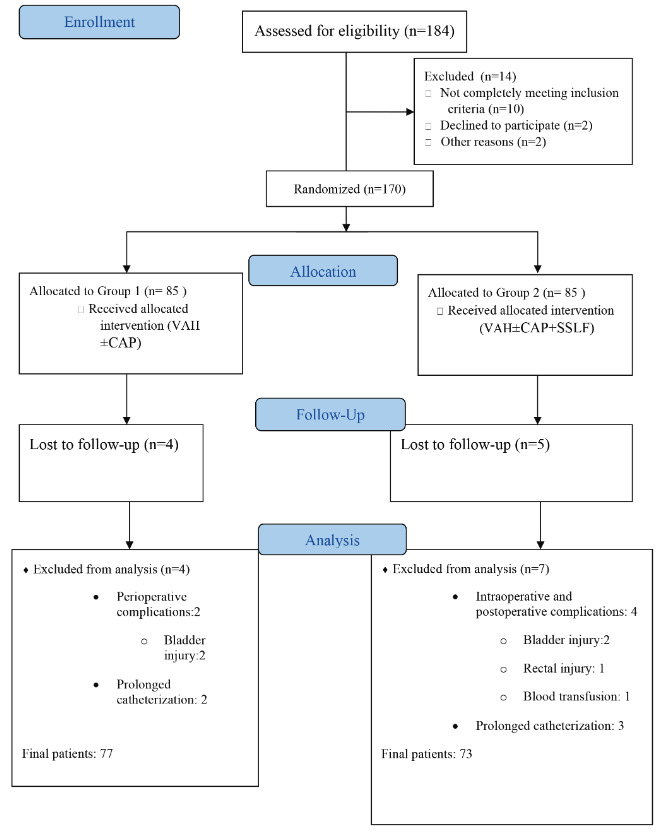
Patient flow chart.

Mean age, gravidity, parity, BMI, menopausal status, and preoperative POP-Q scores, grade 1 and grade 2-3 cystocele and rectocele frequencies were similar between the 2 groups (Table 1). Operative times were significantly longer in Group 2 (P = 0.02). Change in hemoglobin and hematocrit values, and postoperative voiding dysfunction rates were similar between groups (Table 2). During follow-up period, de novo SUI developed in 9 patients (11.6%) of Group 1, and in 7 patients (9.5%) of Group 2 (P > 0.05). 

**Table 1:** Demographic and clinical data of the study patients.

		Group 1 VAH + CAP* N: 77	Group 2 VAH + CAP + SSLF** N: 73	P value
Age (years)		51.9 ± 8.8	53.8 ± 9.69	0.62
Gravidity		2 (0–6)	3 (0–7)	0.48
Parity		2 (0–4)	3 (0–5)	0.21
BMI*** (kg/m2)		28.7 ± 2.1	31.1 ± 2.7	0.16
Postmenopausal patients		49 (63.6%)	58 (79.4%)	0.06
Preoperative POP-Q**** scores	Aa	–1.3 ± 0.7	–1.1 ± 0.9	0.11
	Ba	2.9 ± 1.8	3.6 ± 2.5	0.09
	C	1.9 ± 2.1	2.2 ± 2.3	0.14
	Ap	–1.4 ± 0.3	–1.6 ± 0.5	0.18
	Bp	–2.2 ± 0.9	–0.9 ± 1.2	0.06
Cystocele grade 1		9 (11.7%)	11 (15.06%)	0.06
Cystocele grade 2-3		68 (88.3%)	62 (84.9%)	0.12
Rectocele grade 1		21 (27.2%)	14 (19.1%)	0.37
Rectocele grade 2-3		56 (72.8%)	59 (81.9%)	0.22
POP stage 2		32 (41.5%)	25 (34.3%)	0.07
POP stage 3		45 (58.5%)	48 (65.7%)	0.09

* Vaginal hysterectomy + Colporrhaphy anterior-posterior. ** Vaginal hysterectomy + Colporrhaphy anterior-posterior + Sacrospinous ligament fixation. *** Body mass index. **** Pelvic organ prolapse quantification score.

**Table 2:** Perioperative data of the study patients.

	Group 1 VAH + CAP* N: 77	Group 2 VAH + CAP + SSLF** N: 73	P value
Operative times	86.2 ± 17.3	129.5 ± 19.7	0.02
Hemoglobin change (gr/dL) (ΔHgb)	1.47 ± 0.49	2.19 ± 0.36	0.08
Hematocrit change (%) (ΔHtc)	5.5 ± 1.2	6.7 ± 1.47	0.14
Residual urine volume (ml)	65.4 ± 47.2	53.4 ± 38	0.36
Uterine weight (gr)	166.1 ± 48.5	132.2 ± 33.6	0.73
Postoperative voiding dysfunction	8 (10.3%)	6 (8.2%)	0.21
De novo SUI*** occurrence in the first 6 months	7 (9.1%)	5 (6.8%)	0.56
De novo SUI occurrence in 6–12 months	2 (2.5%)	2 (2.7%)	0.43
De novo SUI occurrence in 12–24 months	-	-	NA
Cumulative de novo SUI in 24 months	9 (11.6%)	7 (9.5%)	0.11
Total vaginal length change (ΔTVL+) 0–24 mos	1.4 ± 0.3	2.1 ± 0.6	0.22
Urethrovaginal prolapse change (ΔAa+) 0–24 mos	2.1 ± 0.4	2.8 ± 0.4	0.16
Postoperative cuff prolapse in 24 months	5 (6.4%)	1 (1.3%)	0.01
Additional cuff prolapse surgery	3 (3.8%)	-	NA

* Vaginal hysterectomy + Colporrhaphy anterior-posterior. ** Vaginal hysterectomy + Colporrhaphy anterior-posterior + Sacrospinous ligament fixation. *** Stress urinary incontinence. + The values are calculated using preoperative and 24th month postoperative values.

## 4. Discussion

POP is an important healthcare problem, particularly affecting the middle aged and elderly women [21]. Surgical treatment of POP without antiincontinence surgery might cure SUI in some cases, whilst it might also unmask a previously occult SUI in others [22]. Occult SUI is very common in women with POP, and preoperative testing is essential to detect this condition [23]. A prolapse reduction urodynamic testing was performed for all cases in the present study, and only women without SUI and occult SUI were selected for allocation into either 1 of the study groups.

In the present study, we aimed to determine if the addition of SSLF technique to VAH and CAP could result in increased de novo SUI in patients with POP. A long follow-up period (24 months) was chosen to assess the long-term effects of this additional procedure. The rates of de novo SUI in women without occult SUI in the present study was 11.6% in Group 1 (VAH + CAP), and 9.5% in Group 2 (VAH + CAP + SSLF) (P > 0.05) (Table 2). These rates are compatible with the relevant literature, although some studies have reported very high rates, approaching 51% [4–11]. These differences might be a consequence of variable patient selection criteria among different studies, as well as the applied surgical techniques. Meticulous preoperative investigation for occult SUI in the present study is believed to be the most important reason for the relatively low rate of de novo SUI. 

Correction of apical prolapse is especially important in POP surgery to prevent future vaginal cuff prolapse [24]. Uterosacral ligament suspension, SSLF, abdominal open and laparoscopic sacral colpopexy are mainly utilized surgical procedures to support the vaginal apex [11, 18]. Each of these procedures carries their own benefits and risks. Careful patient selection and implementing the appropriate surgical procedure that the surgeon is experienced on would increase the success rates and minimize complications. In a previous long term study, both uterosacral ligament suspension (ULS) and SSLF had a relatively high surgical failure rate in 5 years (61.5% and 70.3%, respectively) [25]. However, although anatomic failure rates were high, subjective complaints remained low on the long term follow-up [25]. A modified extraperitoneal uterosacral ligament suspension technique was applied to all patients in Group 1. In Group 2, SSLF was added as an adjunctive intervention. At 24 months, vaginal cuff prolapse rates and the need for additional surgery were higher in Group 1 (Table 2). The SSLF group (Group 2) had significantly less anatomical failure. 

The exact underlying mechanism and risk factors of de novo SUI occurrence is yet to be determined. In a previous study by Alas et al., the authors hypothesized that de novo SUI occurrence could be more common in apical suspension procedures, and recommended counseling every case about the potential risks [11]. The probable effect on SUI development is posterior and apical displacement of the vaginal axis. Moreover, we believe that unilateral displacement of the vaginal apex could further contribute to ureterovesical angle impairment and result in de novo SUI. However, as the interactions between the pelvic organs are very complex in nature, the exact anatomical and physiopathological determinants should be further investigated. In the present study, although the anatomical position of the urethrovesical angle was changed in the SSLF group, no significant effect on incontinence rates was observed. 

Lensen et al. retrospectively analyzed 907 cases of POP surgery, and reported that de novo SUI occurred in 22% of the cases [3]. The authors of this study aimed to determine the potential risk factors, and reported that the highest risk factor for postoperative SUI was preoperative SUI. The authors further concluded that higher BMI and chronic pulmonary obstructive disease (COPD) were important risk factors for postoperative SUI occurrence [3]. Jelovsek et al. conducted a study to define a model to predict de novo SUI in women undergoing POP surgery [26]. The model included patient age, number of previous vaginal births, urine leakage associated with urgency, history of diabetes, BMI, preoperative stress test result, and placement of a midurethral sling were used to calculate the predicted probability of an individual developing de novo SUI. The authors stated that the model was a valid option to facilitate decision making for the addition of a concomitant SUI procedure in women being operated for POP [26]. In a recent meta-analysis, data from19 randomized controlled trials (RCT) including 2717 women were analyzed. The authors concluded that, concominant addition of SUI surgery (midurethral sling or Burch procedure) probably decreased postoperative SUI to some extent. However, as the results from the analyzed studies were conflicting, the authors recommended that it could be feasible to postpone the antiincontinence procedure. In the present study, de novo SUI onset was exclusively observed within the initial 12-month follow-up period. 

To the best of our knowledge, the present study is one of the first studies that compare the outcomes of VAH + CAP and VAH + CAP + SSLF in terms of de novo SUI development over a fairly long follow-up period. An additional strong point of the present study was the prospective randomized design. In conclusion, it was demonstrated that the addition of SSLF to VAH + CAP is a feasible option which minimizes the risk of anatomical failure, without increasing de novo SUI rates. Further studies with larger sample sizes are needed to support this conclusion. 

## Conflict of interest

The manuscript authors report no conflicts of interest.

## Informed consent

The study protocol received institutional review board approval (Approval number: KAEK 2016/2, Clinical Trials: NCT03221725). All participants provided informed consent in the format required by the relevant authorities and boards. 
